# Integrated analysis of multi-omics and fine-mapping reveals a candidate gene regulating pericarp color and flavonoids accumulation in wax gourd (*Benincasa hispida*)

**DOI:** 10.3389/fpls.2022.1019787

**Published:** 2022-09-26

**Authors:** Lingling Xie, Jin Wang, Feng Liu, Huoqiang Zhou, Ying Chen, Luzhao Pan, Wei Xiao, Yin Luo, Baobin Mi, Xiaowu Sun, Cheng Xiong

**Affiliations:** ^1^ College of Horticulture, Hunan Agricultural University, Changsha, China; ^2^ Vegetable Research Institute, Hunan Academy of Agricultural Sciences, Changsha, China; ^3^ College of Horticulture, Nanjing Agricultural University, Nanjing, China

**Keywords:** wax gourd, flavonoid biosynthesis, fine-mapping, *BhiPRR6*, pericarp color

## Abstract

Wax gourd (*Benincasa hispida*), a popular fruit of the Cucurbitaceae (cucurbits) family, contains many nutrients with health benefits and is widely grown in China and other tropical areas. In this study, a wax gourd mutant *hfc12* with light-color pericarp was obtained through ethane methylsulfonate (EMS) mutagenesis. Integrative analysis of the metabolome and transcriptome identified 31 differentially accumulated flavonoids (DAFs; flavonoids or flavonoid glycosides) and 828 differentially expressed genes (DEGs) between the *hfc12* mutant and wild-type ‘BWT’. Furthermore, BSA-seq and kompetitive allele specific PCR (KASP) analysis suggested that the light-color pericarp and higher flavonoid content was controlled by a single gene *BhiPRR6* (*Bhi12M000742*), a typical two-component system (TCS) pseudo-response regulator (PRR). Genetic analysis detected only one nonsynonymous mutation (C-T) in the second exon region of the *BhiPRR6*. Weighted correlation network analysis (WGCNA) identified the downstream target genes of *BhiPRR6*, probably regulated by light and were intermediated in the regulatory enzyme reaction of flavonoid biosynthetic pathway. Thus, these results speculated that the transcription factor *BhiPRR6*, interacting with multiple genes, regulates the absorption of light signals and thereby changes the pericarp color and synthesis of flavonoids in wax gourd.

## Introduction

Wax gourd [*Benincasa hispida* (Thunb.) Cogn., 2n = 2x = 24] is an important vegetable of the Cucurbitaceae family widely grown in the tropical and subtropical regions of the world (Xie et al., 2019). The plant bears large fruit with a dark-green pericarp and has high nutritional values, such as proteins, carbohydrates, vitamins and minerals (Sreenivas et al., 2011). As an important commodity value feature, peel color is a highly variable trait controlled by a relatively complex genetic mechanism, and mainly depends on the content and composition of flavonoids (Li et al., 2013).

As one of the major components of wax gourd, the flavonoids have attracted breeders and consumers for their ability to improve metabolic disorders and antiangiogenic and anticancer effects (Zoratti et al., 2014). Besides increasing the nutritional value of the fruit, flavonoids act as indicators of fruit quality also in wax gourd (Imran et al., 2019). Flavonoids are polyphenolic secondary metabolites of plants with different functions and include flavonols, flavones, flavanones, flavan-3-ols, isoflavones, and anthocyanins. They are generally found in plant-based foods, such as fruits, vegetables, beans, and tea, in the bound (flavonoid glycosides) or free form (flavonoid aglycones) (Azuma et al., 2012). Several studies have shown flavonoids have high nutritional functions in the reaction of the organism such as the antioxidant, anti-inflammatory, and antitumor properties (Jaakola and Hohtola, 2010). Notably, flavonoids play a central role in fruit quality and economic value; they influence the color, aroma, astringency, and antioxidant properties of fruits. For example, flavonols provide photoprotective effects, flavan-3-ols (precursors of proanthocyanidins) influence fruit flavor (Sudheeran et al., 2021), and the degree of co-pigmentation of anthocyanins and flavanones affected the fruit color (Baranac et al., 1997). Therefore, it is necessary to understand the factors that regulate the biosynthesis of wax gourd flavonoids, which may help develop and produce improved wax gourd varieties.

Flavonoids are synthesized through the phenylpropanoid and flavonoid pathways. In recent years, the biosynthetic pathway of flavonoids and the structural genes encoding the flavonoid biosynthetic enzymes have been extensively studied (Martens et al., 2010). In the early stages of the flavonoids biosynthetic pathway, the phenylalanine ammonia-lyase (PAL), chalcone synthase (CHS), chalcone isomerase (CHI), flavanone 3-hydroxylase (F3H), and coumadin CoA ligase (4CL) were flavonoid biosynthetic enzymes to produced p-coumaroyl-CoA (precursor substance) (Winkel-Shirley, 2002). Naringenin, catalyzed by chalcone synthase (CHS) and chalcone isomerase (CHI), is the core intermediate that leads to different flavonoid subclasses from each branch in the pathway (Jaakola, 2013). The biosynthesis of anthocyanins is a major branch of the flavonoid pathway, regulated by dihydroflavonol 4-reductase (DFR), UDP-glucose: flavonoid 3-glucosyltransferase (UFGT), and anthocyanin synthase (ANS) (Almeida et al., 2007). Anthocyanin reductase (ANR) and leucoanthocyanidin reductase (LAR) play central roles in proanthocyanidin biosynthesis (Shi et al., 2020). Meanwhile, flavonol is synthesized from dihydroflavonols by flavonol synthase (FLS). Flavones use flavanones as substrate and are formed by flavone synthase (FS) (Tomás-Barberán et al., 2001).

In addition to the structural genes encoding the enzymes of the flavonoid pathway, transcriptional factors regulate the expression of genes encoding these enzymes in each synthesis step. Many transcription factors have been found to regulate the flavonoid pathway (Yan et al., 2021). In general, the transcription factor R2R3-MYB interacts with bHLH (MYC-like basic helix-loop-helix) protein to form a complex with WD40 protein (MBW complex), which regulates the coordinated transcription of structural genes of the flavonoid biosynthetic pathway (Hichri et al., 2011). In apple (*Malus × domestica* Borkh.), *MdMYB73* regulates the accumulation and vacuolar acidification of anthocyanins by directly activating the vacuolar transporters *(MdVHA-B1*, *MdVHA-E*, *MdVHP1*, and *MdtDT*) (Hu et al., 2017). In tomato (*Solanum lycopersicum*), *SlMYB14* was involved in the regulation of flavonoid biosynthesis, and played a role in maintaining plant active oxygen homeostasis (Li et al., 2021). In mango (*Mangifera indica* L.), *MiMYB1* acts as a critical regulator of anthocyanin biosynthesis and regulates the light-dependent red coloration (Kanzaki et al., 2020). *PpMYB17*, a bHLH or WD40 cofactor in the MBW complex, activates the structural genes *PpCHS*, *PpCHI*, *PpF3H*, and *PpFLS* involved in flavonoid biosynthetic pathway, and was positively regulates flavonoid biosynthesis in pear (*Pyrus* spp.) fruits (Premathilake et al., 2020). Studies have also reported other transcription factors that regulate flavonoid synthesis. For example, *SQUAMOSA MADS-box* gene, a critical regulatory factor in bilberry (*Vaccinium myrtillus*) fruits, is involved in anthocyanin biosynthesis (Jaakola et al., 2019). In muskmelon (*Cucumis melo*), a *CmKFB* gene negatively regulates flavonoid accumulation (Feder et al., 2015). In recent years, metabolome and transcriptome analysis have shown that *R2R3-MYB*, *bHLH51* and *WRKY23* were candidate key transcription factors that regulate the biosynthesis of flavonoids in cucumber peel (Chen et al., 2021). In wax gourd, a single gene locus on chromosome five controlled peel color (Jiang et al., 2015). However, the transcription factors that regulate the flavonoids synthesis of wax gourd have not been reported.

Ethyl methanesulfonate (EMS), as a chemical mutagen, has been widely used in plant mutagenesis breeding and functional genome research due to its high mutagenesis efficiency, easy operation and wide mutagenesis range. Therefore, EMS mutagenesis can not only obtain deletion or gain-of-function mutants, but also help to understand the role of specific amino acid residues in protein function (Yuan et al., 2014). BSA (Bulked Segregant Analysis), also known as mixed grouping analysis or cluster segregation analysis, is very suitable for analyzing mutants induced by EMS mutagenesis. Two materials with significant differences are used as parents, and a segregated population is constructed by hybridization. About 30 plants with extreme phenotypes were selected to construct two mixed pools. The difference region obtained by sequencing and analysis of the two mixed pools was the candidate region, and the target gene may exist in this region (Kurlovs et al., 2019). In this study, EMS mutagenesis generated a wax gourd mutant *hfc12* with light peel color and high flavonoid content. The transcriptome sequencing (RNA-seq) and the metabolome (liquid chromatography with tandem mass spectrometry, LC–MS/MS) of the *hfc12* mutant and WT ‘BWT’ were analyzed to understand the regulation of flavonoid biosynthesis. A single gene *BhiPRR6* (pseudo-response regulator, *Bhi12G000742*) located on the chromosome 12 was fine-mapped by genetic BSA-seq analysis, which regulated the flavonoid biosynthesis in wax gourd. WGCNA was carried out to explore the correlation of the candidate gene with target genes in regulating the flavonoid pathway. To conclude, this study clarified the role of *BhiPRR6* gene in wax gourd fruit flavonoid biosynthesis and revealed its regulatory network. The findings will provide a theoretical basis of molecular genetic improvement in wax gourd.

## Materials and methods

### Plant materials, EMS mutagenesis, and mutant analysis

Wax gourd wild-type (WT) inbred line “BWT” was treated by 0.8% EMS to construct a mutant library, and a wax gourd mutant *hfc12* with high flavonoid content was generated. The ‘BWT’ and *hfc12* plants as parents to obtain an F_1_ population and then were further self-crossing to establish the F_2_ mapping population (486 individuals), including 361 ‘BWT’ and 125 *hfc12* individuals, respectively. At the same time, F_1_ and *hfc12* were backcrossed to obtain 238 individuals BC_1_ population. All wax gourd varieties were planted in the Changsha experimental station (N 28°11′49″, E 112°58′429″) of the Hunan Vegetable Research Institute of Agricultural Science at the Apr. of 2018, Changsha, China. The flavonoid content, carotenoid content, chlorophyll a (Ca), and chlorophyll b (Cb) of the parents and F_2_ population were determined at the same time. The flavonoid contents of wax gourd pericarp were determined by colorimetry using the Plant Flavonoids test kit (A142-1-1, Nanjing Jiancheng Bioengineering Institute). The carotenoid content, chlorophyll a (Ca), chlorophyll b (Cb) was determined by visible spectrophotometry using a Plant Carotenoid detection kit (BC4330, Beijing Solarbio Science & Technology Co., Ltd.). The segregation ratios in the F_2_ and BC_1_ populations were analyzed with the Chi-square (χ^2^) goodness of fit test using R.

### Transcriptome analysis

Total RNA was extracted from the frozen wax gourd pericarp of parents ‘BWT’ and *hfc12* collected at 40 days after pollination (DAP, 40DAP mature stage: Pericarp color change stage) were using TransZol Kit (TransGen Biotech, Inc., Beijing, China). The libraries (200–250 bp) of each sample were generated and sequenced on an Illumina HiSeq™ X-Ten platform for paired-end reads (BGI, Shenzhen, China). The quality of reads obtained was checked using the Fastqc program (Brown et al., 2017). Trimmmatic (v0.36) was used to trim the adapter sequences and remove low-quality reads (Bolger et al., 2014). The filtered reads were mapped to the wax gourd reference genome (*B. hispida* var.* B227*) (Xie et al., 2019) using the Salmon (v1.2.0) tool (Patro et al., 2017). TPM (Transcripts Per Million) was used to quantify the gene/transcript levels. DESeq2 was used to identify the DEGs with the criteria of |log2Fold Change| ≥ 1 and a false discovery rate (FDR) < 0.05 (Love et al., 2014). Gene Ontology (GO) was annotated using Plant Transcriptional Regulatory Map online software (http://plantregmap.gao-lab.org/), and Kyoto Encyclopedia of Genes and Genomes (KEGG) pathway enrichment was analyzed by Kobas3.0 online software. Both GO and KEGG were conducted using clusterProfiler (R, v3.12) with the corrected *P*-value ≤ 0.05 (Yu et al., 2012). The expression level of the genes in the whole tissue period were obtained from CuGenDB (http://cucurbitgenomics.org/) (Xie et al., 2019).

### Metabolite profiling

Samples were extracted from the frozen wax gourd pericarp of ‘BWT’ and *hfc12* collected at 40 DAP with each three biological replicates. The biological samples were placed in a freeze dryer (Scientz-100F) for vacuum freeze drying. Using a grinder (MM 400, Retsch) with a zirconia bead for 1.5 min at 30 Hz. Using ultra-performance liquid chromatography (UPLC; SHIMADZU Nexera X2) and tandem mass spectrometry (MS/MS) (Applied Biosystems 4500 QTRAP) to analyzed. A widely targeted metabolomics method was used based on the self-built database MWDB (Metware Biotechnology Co., Ltd. Wuhan, China) (http://www.metware.cn/). The metabolites were qualitatively analyzed based on the secondary spectrum information. The isotope signal is removed during analysis, including repeated signals of K^+^, Na^+^, NH4^+^, and the repeated signals of fragment ions that are other larger molecular weight substances.

Unsupervised PCA was performed using the statistics function prcomp of the R statistical software (www.r-project.org). The hierarchical cluster analysis (HCA) results of samples and metabolites were presented as heatmaps with dendrograms. Pearson correlation coefficients (PCC; *r*) between the samples were calculated using the cor function in R and presented as a heatmap. Both HCA and PCC were carried out using the pheatmap (R, v1.0.12). For HCA, normalized signal intensities of metabolites (unit variance scaling) were visualized as a color spectrum. Significantly regulated metabolites between ‘BWT’ and *hfc12* were determined by VIP ≥1 and an absolute Fold change ≥ 2 or Fold change ≤ 0.5. VIP values were extracted from the orthogonal projections to latent structures discriminant analysis (OPLS-DA) result, with score plots and permutation plots, generated using MetaboAnalystR (R, v3.0) (Pang et al., 2020). The data were log-transformed (log_2_) and mean-centered before OPLS-DA. A permutation test (200 permutations) was performed to avoid overfitting. Identified metabolites were annotated using KEGG Compound database (http://www.kegg.jp/kegg/compound/), annotated metabolites were then mapped to KEGG Pathway database (http://www.kegg.jp/kegg/pathway.html). Pathways with significantly regulated metabolites mapped to were then fed into metabolite sets enrichment analysis (MSEA).

### Bulked segregant analysis RNA sequencing and linkage analysis

The DNA samples of the parental lines ‘BWT’ and *hfc12* were prepared as two pools for sequencing with each three biological replicates. From the F_2_ population, 25 pericarps of wax gourd at 40 DAP for each plant with extremely high flavonoid content and 25 extremely low flavonoid content individuals were selected to construct two separation pools. In addition, DNA samples were extracted from individual plants in each pool and mixed in equimolar amounts to form wild-type mixed pools (low flavonoid content) and mutant mixed pools (high flavonoid content) for constructing genomic libraries. Four cDNA libraries (dominant mixed pool, recessive mixed pool, and two parent pools) were constructed using Truseq Nano DNA HT sample preparation kit (Illumina, USA). These four libraries were sequenced and evaluated on the Illumina HiSeq 4000 platform (Changsha, China) to generate 2×150 bp paired-end reads with an insert size of about 350 bp. The sequencing depth of the two mixed pools were 25×, and the sequencing depth of the two parent pools were 10×. Fastqc was used to evaluate the quality of the reads, and Trimmomatic to remove the adaptors and low-quality reads (Bolger et al., 2014; Brown et al., 2017). Hisat2 was used to map the clean reads to the wax gourd reference genome. SAMtools (v0.1.18) software was used to perform SNP calling with 90% loci depth coverage (Ramirez-Gonzalez et al., 2012). Further, Burrows-Wheeler Aligner (BWA) was used to align with the reference genome (*B. hispida* var.* B227*), and GATK (Genome analysis toolkit, v4.0) was used to detect SNPs and InDels. ANNOVAR was used to annotate the SNPs and InDels based on the GFF3 file of the wax gourd reference genome. In addition, the SNP-index algorithm was used to identify candidate regions of the genome related to flavonoid content, and calculated the difference in allele frequency between large-capacity pools (Takagi et al., 2013). Δ (SNP index) was the difference in SNP-index between the dominant mixed pool and the recessive mixed pool. The points with an SNP/InDel index less than 0.3 were filtered out of both pools. According to the SNP △(SNP-index) >0.5 and Euclidean distance (ED), the candidate regions related to the flavonoid content are selected with 95% confidence. Join Map software (v4.0) was used to construct the genetic linkage map with five SNPs as the window, and two SNPs as the step size.

### Fine-mapping analysis

Mapping function was used to calculate the genetic map distance (cM) of the F_2_ population 486 individuals from the recombination frequency (Voorrips, 2002). Based on the reference genome, Kompetitive Allele Specific PCR (KASP) was used to accurately determine SNPs and InDels biallelic genes at specific sites (Semagn et al., 2014). The CTAB method was used to extract DNA from all peel samples of 486 F_2_ individuals for KASP analysis. The quality and quantity of the extracted DNA were verified by agarose gel electrophoresis and NanoDrop ND-2000 spectrophotometer, and the primers were designed by Primer5 (**Table S4**). The 200 bp sequence upstream and downstream of the SNPs were used to design the KASP primers, and the SNPs were typed based on the specific matching of primer end bases. KASP method and mix were to refer to PARMS SNP detection reagent manual (Gentides Biotech Co., Ltd (Wuhan) Synthesis), and were carried out on LightCycle^®^ 96 Real-Time PCR System (Roche, Basel, Switzerland) in a 10μL reaction mixture. The selected genes and their primer sequences were listed in **Table S4**.

### Real time fluorescence quantitative PCR

The qRT-PCR method was used to quantify the transcript abundance in the 40 DAP pericarp of the wax gourd plants. Reverse transcription was carried out using the HiScript^®^IIQ RT SuperMix for qRT-PCR (+gDNA wiper) (Vazyme Biotech Co. Ltd, Piscataway, NJ, United States). Primers were designed using Primer3 (http://bioinfo.ut.ee/primer3-0.4.0/), with the PCR product size set at a range of 80–150 bp. The qRT-PCR was carried out on LightCycle^®^ 96 Real-Time PCR System (Roche, Basel, Switzerland) in a 20μL reaction mixture by using ChamQ Universal SYBR qPCR Master Mix (Vazyme Biotech Co. Ltd, Piscataway, NJ, United States). Three biological replicates and three technical replicates were maintained. *Actin* was used as the reference gene. The 2^-ΔΔCt^ method was used to calculate the relative expression level of the genes. The selected genes and their primer sequences were listed in **Table S4**.

### Correlation analysis

The WGCNA package in R language was used to construct a co-expression network and analyze the correlation of gene modules. RNA-seq FPKM data were used to perform WGCNA analysis (Langfelder and Horvath, 2008). The DEGs along DAFs with PCC ≥0.90 or ≤0.90 were selected for subsequent WGCNA with default parameters. Cytoscape was used to generate style and statistical a co-expression plot (Smoot et al., 2011). The DEGs in co-expression network were also subjected to GO and KEGG enrichment analysis, same method as above Transcriptome analysis of Materials and Methods part.

## Results

### Phenotypic characterization of ‘BWT’ and *hfc12*

**Figure 1:**
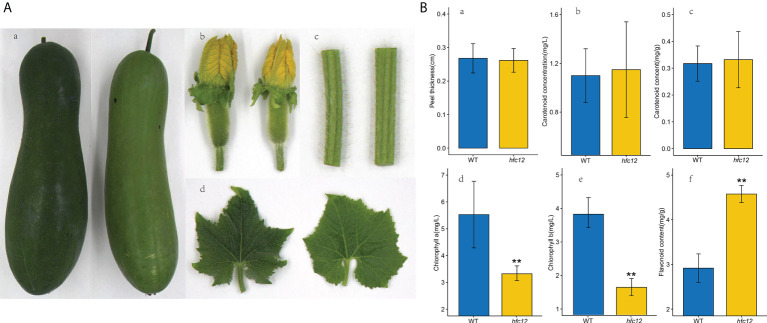
Phenotypic characterization and physiological indicators of WT and *hfc12*. **(A)** Phenotypic characterization of fruits at 40 DAP (a), flowers (b), stems (c), and leaves (d) of WT (left) and *hfc12* (right). **(B)** Physiological indicators including peel thickness (a), carotenoid concentration (b), carotenoid content (c), chlorophyll a content (d), chlorophyll b content (e), and flavonoid content (f) of ‘BWT’ (left) and *hfc12* (right). Asterisks indicate significance according to the t-test (**P< 0.01).

EMS was used to mutate the WT wax gourd variety ‘BWT’ to construct a mutant library. After multiple generations of screening, a mutant *hfc12* plant with light-pericarp color was obtained. Phenotypic analysis revealed that the F_2_ generation fruit peel of *hfc12* at 40 DAP was light green compared with the ‘BWT’ (**Figure 1A**). However, the *hfc12* color of the stem, leaves, and flowers was not significantly different from that of the ‘BWT’; no significant difference was observed in the peel thickness also. The concentration of polyphenols and other compounds in the fruits of the *hfc12* and ‘BWT’ was analyzed. The flavonoid content (4.71 mg/g) in the *hfc12* was significantly higher than that of ‘BWT’ (2.85 mg/g). Besides, the content of chlorophyll a (5.52 mg/L) in the ‘BWT’ was significantly higher than that of *hfc12* (3.38 mg/L), and the content of chlorophyll b (3.83 mg/L) in the ‘BWT’ was significantly higher than that of *hfc12* (1.65 mg/L). No significant difference was observed between the *hfc12* and the ‘BWT’ in carotenoid concentration and content (**Figure 1B**).

### Identification and functional analysis of DEGs

**Figure 2:**
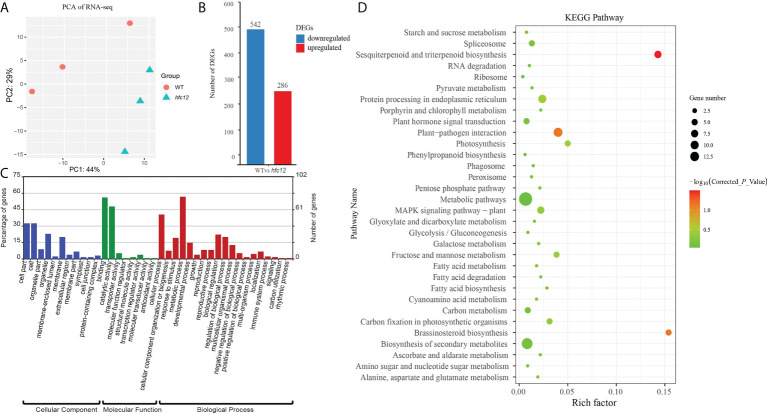
RNA-seq transcriptome analysis of WT and *hfc12*. **(A)** PCA plot of DEGs between ‘BWT’ and *hfc12*. **(B)** Differentially expressed genes (DEGs) at 40 DAP of WT and *hfc12*. **(C)** GO enrichment analysis of DEGs. Blue bars for cellular component, green bars for molecular function, and red bars for biological process. **(D)** KEGG analysis of DEGs.

To further understand the flavonoid response patterns of ‘BWT’ and *hfc12* at the transcriptional level, genes related to higher flavonoid levels were identified from wax gourd. Transcriptome sequencing (RNA-seq) was performed using the *hfc12* and ‘BWT’ fruit frozen pericarp samples collected at 40 DAP. After deleting the adaptors and low-quality reads from the raw data, 55.05 Gb clean data were generated from the six samples. The number of reads per sample ranged from 20.27 million to 28.85 million, with an average of 22.80 million (**Table S1**). Approximately 95% of the clean reads were mapped to the wax gourd (*B. hispida* cv. B227) reference genome. Furthermore, the correlation coefficient and principal component analysis (PCA) suggested a high degree of similarity between the biological repeats (**Figure 2A**). A total of 828 DEGs were identified between ‘BWT’ and *hfc12* at 40 DAP, including 286 upregulated and 542 downregulated genes (**Figure 2B**).

GO analysis was performed to identify the primary biological functions of all the DEGs between ‘BWT’ and *hfc12* with the corrected *P*-value < 0.05. All DEGs were assigned GO terms of the three major categories: cellular component, molecular function, and biological process (**Figure 2C**). DEGs were primarily assigned in cell part, cell, organelle, and membrane in the cellular component category. In the molecular function category, catalytic activity and binding were significantly assigned. In the biological process category, 56% of DEGs were primarily assigned in the metabolic processes (GO: 0008152), specially. Cellular process, response to stimulus, biological regulation, and regulation of biological process were also highly assigned in the biological process category.

Furthermore, KEGG pathway analysis revealed that DEGs were associated with 32 common metabolic and biological pathways (**Figure 2D**). DEGs were preferably enriched in the metabolic pathways, biosynthesis of secondary metabolites, plant hormone signal transduction, protein processing in endoplasmic reticulum. Besides, DEGs were significantly enriched (− log_10_(Corrected_*P*_Value) >0.1) in sesquiterpenoid and triterpenoid biosynthesis, and brassinosteroid biosynthesis Photosynthesis, MAPK signaling pathway - plant, fructose and mannose metabolism, and carbon fixation in photosynthetic organisms were also enriched.

### Identification and enrichment analysis of DAFs in the fruits

**Figure 3:**
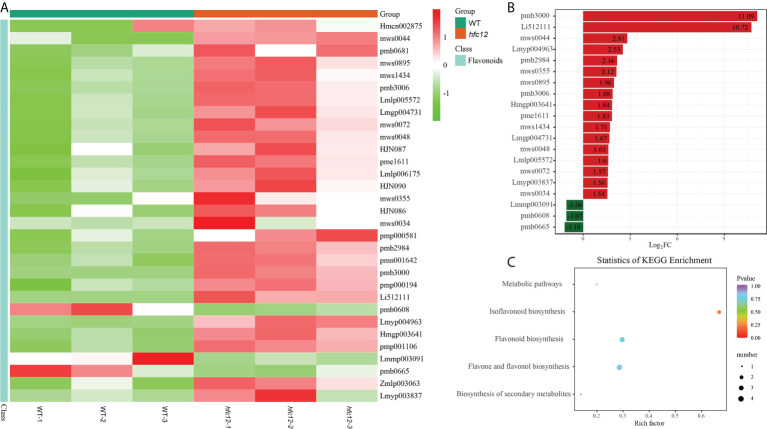
Metabonomic analysis of WT and *hfc12*. **(A)** Heatmap of DAFs between WT and *hfc12*. The columns represented samples, and the rows represented the differential metabolites. The differential metabolite cluster tree was shown on the left side of the plot. The different colors indicated the values obtained after standardization of the relative content (red represents high content, green represents low content). **(B)** Bar chart of DAFs. **(C)** KEGG enrichment scatter plot of DAFs.

TRAP-MS/MS was performed to compare the differences in flavonoid composition between the ‘BWT’ and *hfc12* wax gourd fruit frozen pericarp samples collected at 40 DAP (**Figure S1A**). A total of 138 flavonoids were detected in all samples, including 29 flavonoid carbonosides, 26 flavonols, 51 flavonoids, 13 dihydroflavones, two chalcones, and eight isoflavones, eight kinds of tannins and one kind of proanthocyanidins (**Table S2**). Among them, a total of 31 DAFs were identified between ‘BWT’ and *hfc12* (fold change ≥ 2 or fold change ≤ 0.5, and variable importance in projection (VIP) ≥1), of which 28 were upregulated and three were downregulated. These DAFs were either flavonoids or flavonoid carboglycosides. Twelve flavonoids were identified in DAFs (**Figure 3A**). Besides, one chalcone, two flavanols, three flavonols, three isoflavones, three dihydroflavonol, and seven flavonoid carbonosides were also among the DAFs. Of which, chrysoeriol-7-O-(6’’-acetyl) glucoside and isorhamnetin-3-O-(6’’-acetylglucoside) were the highest upregulated in the *hfc12*, with a log_2_FC value of 11.09 and 10.72, respectively. Orientin-7-O-glucoside, chrysoeriol-7-O-(6’’-malonyl) glucoside, and quercetin-3-O-(4’’-O-glucosyl) rhamnoside were the three downregulated metabolites in the *hfc12*, with a log_2_FC value of -1.18, -1.07, and -1.06, respectively (**Figure 3B**).

Furthermore, KEGG enrichment analysis revealed that the DAFs were enriched with flavone and flavonol biosynthesis (ko00944), flavonoid biosynthesis (ko00941), isoflavonoid biosynthesis (ko00943), and secondary metabolite biosynthesis pathways (ko01110). These observations indicated that the *hfc12* plant have mutations in the flavonoid metabolism pathway, probably affecting fruit development and peel color changes (**Figure 3C**).

### Mapping of wax gourd *hfc12*

The F_2_ (n = 486) population isolated from the cross between the WT ‘BWT’ and *hfc12* mutant the was used for genetic analysis and primary mapping. Genetic population analysis and chi-square test revealed that the low flavonoid content (361 individuals, dark-green pericarp) and the high flavonoid content (125 individuals, light-green pericarp) traits of the F_2_ generation were separated at a ratio of 3:1, and that of the backcross population at a ratio of 1:1 (**Table 1**), consistent with the Mendelian independent genetic rules. These observations indicated that a single recessive nuclear gene controls the trait related to high flavonoid content.

**Table 1:** Genetic analysis and flavonoids content of wax gourd *hfc12*.

Type	Population	WT	*hfc12*	Theoretical ratio	Actual ratio	χ^2^	Dominant/Recessive	WT flavonoids range (mg/g)	*hfc12* flavonoids range (mg/g)	WT flavonoids mean (mg/g)	*hfc12* flavonoids mean (mg/g)
F_2_	486	361	125	3:1	2.9:1	0.13	R	2.36-3.28	3.97-5.84	2.73	4.78
BC_1_	238	116	122	1:1	0.95:1	0.15	R	-	-	-	-

χ_c_^2^ <χ^2^_0.05(1)_=3.84, P>0.05.

Four parental DNA pools (dominant pool, recessive pool, dominant progeny pool, and recessive progeny pool) were constructed, and the WT pool and mutant pool were mixed to construct a genomic library for BSA-seq. Genome-wide BSA-seq using these DNA pools resulted in 67 Gb of data. The two parental lines generated 0.76 million paired-end reads of ‘BWT’ and 0.61 million paired-end reads of *hfc12*. The sequencing depths were 10.47× and 10.05×, and the genome coverage rates were 95.60% and 95.93% in the ‘BWT’ and *hfc12*, respectively. Similarly, the alignment of the dominant sexual progeny pool and recessive progeny pool were 20.01× and 18.92× of sequencing depth, and genome coverage of 95.67% and 95.93%, respectively (**Table S3**). Statistical analysis suggested that the sequencing data were reliable for BSA-seq analysis. A total of 21,548 SNPs were identified between the ‘BWT’ and the *hfc12* libraries (base quality value ≥ 20, mapping quality value ≥ 20, base depth (two F_2_ mixed pools) ≥ 2 and ≤ 60, and base depth (parents) ≥ 2 and ≤ 60). There were two regions on the whole genome of the recessive pool with Δ (SNP index) values above 0.5 and close to 1, which were located in the 25-30 Mb range of chromosome 12, and a total of 456 candidate sites for the target region have been obtained.

### Fine-mapping and candidate gene analysis

**Figure 4:**
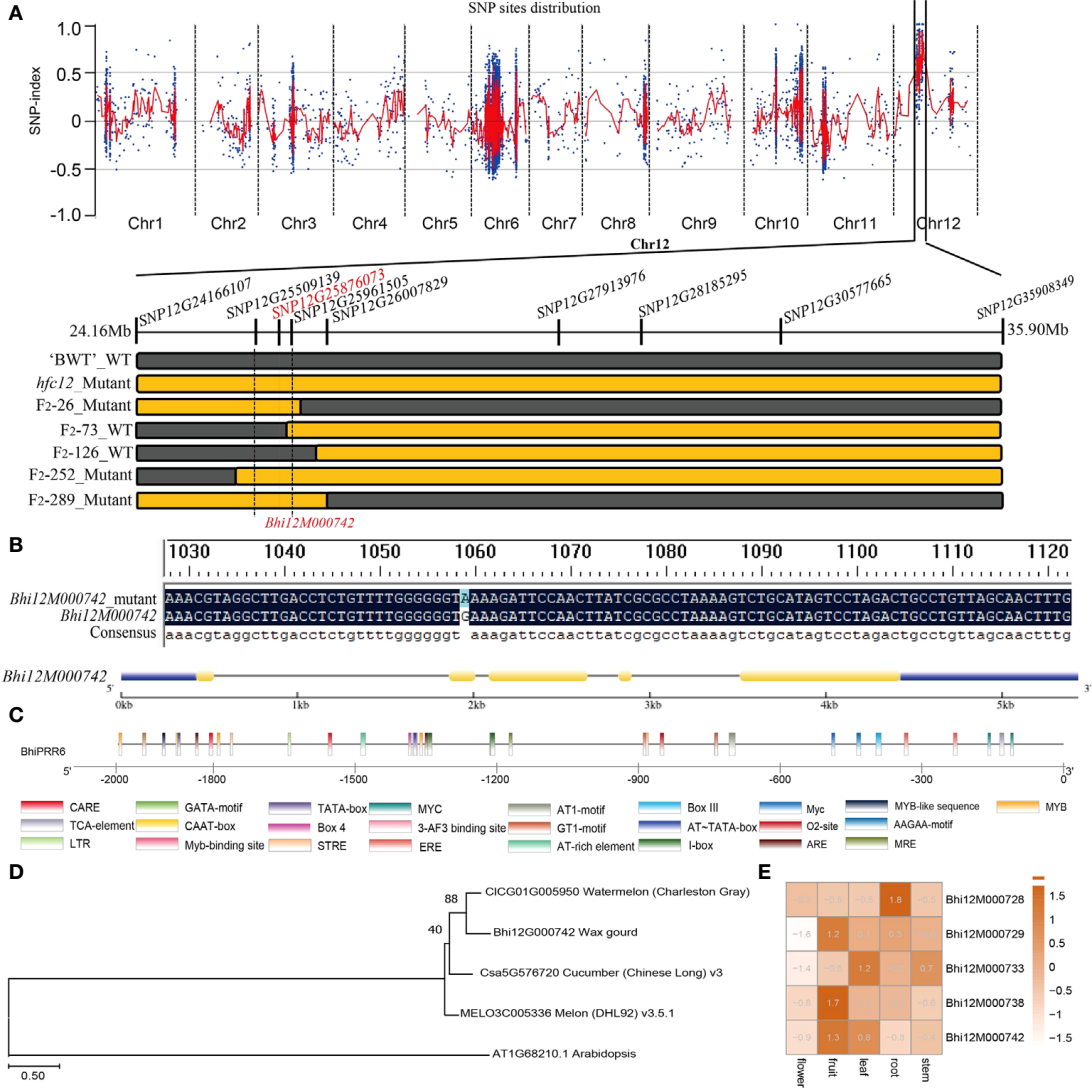
Cosegregation analysis and genetic variation in *BhiPRR4* of wax gourd *hfc12*. **(A)** Gene location and mutation site in wax gourd. BSA-seq generated the SNP distribution Δ(SNP index)] plot; the vertical axis represents the chromosome, and the red vertical line represents the location of the *hfc12* SNP. The highest point shown is the locus 25 Mb to 30 Mb on chromosome 12. Based on F_2_ plants with extreme phenotypes, the genetic map was drawn on the 452.366 kb region (SNP12G25509139 to *SNP12G25961505*). **(B)** *Bhi12G000742* (*BhiPRR6*) mutation of C to T in the SNP on the positive strand (G to A on the negative strand) of the second exon (red line). The yellow rectangle and the black line represent the exons and introns, respectively. **(C)** Schematic representation of *BhiPRR6* promoter 2000 bp upstream of the CDS region. *Cis*-elements are shown in rectangles with different colors. **(D)** Multiple sequence alignment of *BhiPRR6* proteins with watermelon, melon, cucumber, and *Arabidopsis*. **(E)** Different tissue expression levels of genes in the candidate interval.

All F_2_ individuals from ‘BWT’ × *hfc12* were used for KASP molecular markers for genotyping. Based on the genotypes and phenotypes, a total of 78 recombinant plants were identified for further population location. Finally, fine-mapping places the *hfc12* locus in the genomic region, flanked by markers 24.16 Mb (SNP12G24166107) to 35.90 Mb (SNP12G35908349), a 11.7Mb on chromosome 12 (**Figure 4A**). According to the wax gourd reference genome ‘*Benincasa hispida* var. B227’ database, 11 predicted genes were annotated in the candidate region, including nine intron mutations, two 5’ UTR mutations, one 3’ UTR mutation, and one coding sequence (CDS) mutation (**Table S5**). Nine polymorphic SNP markers were further developed from 24.16 Mb to 35.90 Mb region, and the mutation site was finally restricted to the 452.366 kb region (SNP12G25509139 to SNP12G25961505) on chromosome 12 by multiple KASP molecular markers. Within this interval, there were a total of 219 SNPs, and involving 5 genes (**Figure 4E**). However, only one SNP (SNP12G25876073) is located in the gene CDS region and co-segregated with the phenotypes among the F_2_ population, resulting in non-synonymous mutations between WT and *hfc12* (**Figure 4B**). PCR amplification and sequencing identified the SNP-25876073 was located in the second exon of *Bhi12G000742* (*BhiPRR6*). A C-T mutation was detected at 1060 bp of mutant *hfc12* CDS region; CTT was mutated to TTT (+), and Glu (E) to Lys (K) (-) (**Figure 4B**).

Annotation and protein homology comparison found that the candidate gene was a typical two-component response regulator PRR family gene. The analysis identified the gene as *BhiPRR6*, 1802 bp long with five exons and four introns. It had theoretical pI (isoelectric point) and Mw (molecular weight) of 6.88 and 67180.10, respectively, and was located on chromosome 12 (25876073–25877875). The promoter region (2000 bp upstream) of *BhiPRR6* had 26 *cis*-elements, including typical ARE, AT-rich element, ethylene-responsive element (ERE), and salicylic acid-responsive element (TCA-element). It also included various typical MYB *cis*-acting elements such as MRE, MYB, MYB-like sequence, MYC, and MYB-binding site (**Figure 4C**). Besides, *BhiPRR6* was highly homologous to the watermelon (*Citrullus lanatus* subsp. *vulgaris* cv. 97103) *CLCG01G005950*, the muskmelon (*Cucumis melo* L. cv. DHL92) *MELON3C005336* (*CmPRR5*, *LOC103504590*), the cucumber (*Cucumis sativus* L. var. *sativus *cv. 9930) *Csa5G576720*, and the *Arabidopsis AT1G68210* (*APRR6*). The closest homology was found between *BhiPRR6* and the watermelon *CLCG01G005950* gene, with a bootstrap value of 89 (**Figure 4D**). Analysis of the expression levels of five candidate genes in the KASP regions showed that *BhiPRR6* was highly expressed in fruits and leaves (**Figure 4E**).

### Correlation analysis of *BhiPRR6* in flavonoids biosynthetic pathways

**Figure 5:**
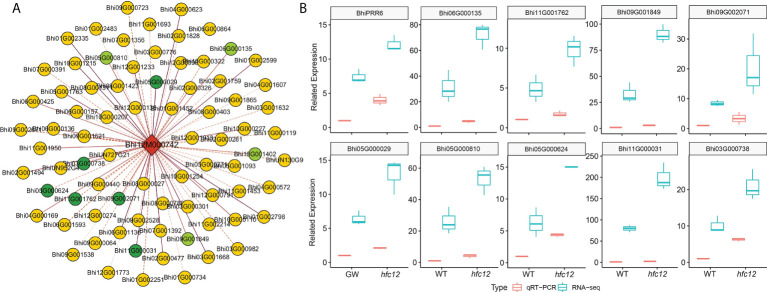
Co-expression regulatory network and expression of *BhiPRR6* and other interacting genes in wax gourd. **(A)** Co-expression network analysis with *BhiPRR6* as hub gene. The light-green circles represent the genes with the highest correlation (Topological overlap matrix, TOM > 0.52). The dark-green circles represent the candidate genes that may regulate the synthesis of flavonoids with *BhiPRR6* (**Table S5**). **(B)** The expression pattern of *BhiPRR6* and ten co-expressed genes by both Quantitative real-time PCR (qRT-PCR) and RNA-seq analysis.

WGCNA was performed using RNA-seq data to study the correlation pattern among the genes in wax gourd. All 971 co-expressed genes with *BhiPRR6* as the hub gene were clustered into eight main modules (marked in different colors) (**Table S6**). Genes in the same module were highly related and co-expressed. The turquoise module had the maximum number of DEGs (258), followed by the blue (148), brown (145), and yellow (144) modules (**Figure S1D**). Co-expression analysis suggested a high correlation between 78 genes and *BhiPRR6*, with a strict edge weight (topological overlap matrix, TOM ≥ 0.30). Among them, F-box family protein (*Bhi11G001950*), glycerol-3-phosphate acyltransferase 1 (GPAT1, *Bhi05G001763*), acyl carrier protein 4 (ACP4, *Bhi02G000477*), and CBL-interacting protein kinase 1 (CIPK1, *Bhi12G001773*) showed a high degree of connectivity, indicating certain biological functions. *Bhi10G001402*, *Bhi06G000135*, *Bhi09G001849*, and *Bhi05G000810* showed the highest co-expression with the hub gene *BhiPRR6* (**Figure 5A**). In particular, co-expressed genes were related to flavonoid biosynthesis, including a structural gene 4CL (4-coumarate-CoA ligase, *Bhi11G001762*), with a TOM value of 0.35. UDP-glucose: flavonoid-3-O-glycosyltransferase (UFGT; *Bhi09G002071*, *Bhi03G000738*, *Bhi05G000029*, and *Bhi05G000624*) and photosystem I chlorophyll a/b-binding protein 6 (LHCA6, *Bhi02G001494*) were also highly co-expressed with *BhiPRR6*. An MYB1R1 (*Bhi11G000031*) also showed a high correlation with *BhiPRR6* involved flavonoid synthesis. A WD40 protein (*Bhi12G000575*) and a 4CL structure protein (*Bhi11G001234*) were also present in this network.

Furthermore, the expression levels of the co-expressed genes were analyzed using RNA-seq data. The analysis divided 79 genes (hub gene and 78 co-expressed genes) into two categories based on the expression pattern. Among them, most co-expressed genes (74 genes) were similar to *BhiPRR6*, with expression levels significantly high at 40 DAP of *hfc12.* In the network, ten candidate co-expressed gene annotations may be related to the flavonoid pathway, with the similar expression pattern of *BhiPRR6* (**Figure 5A**). RNA-seq and qRT-PCR were performed to analyze the transcript level of *BhiPRR6* and the co-expressed genes in the mutant *hfc12* and the WT ‘BWT’ parent individuals. As shown in **Figure 5B**, the expression level of *BhiPRR6* was highest in *hfc12*, two times more than that in WT. At the same time, ten co-expressed genes closely related to *BhiPPR6* were basically the same as their expression forms, and they were all highly expressed in the mutant *hfc12*. Of which, the highest expression was the *Bhi09G001849* gene, and its expression in the *hfc12* was about four times that of the ‘BWT’. The qRT-PCR results were consistent with the RNA-seq results, with an R^2^ above 0.80.

## Discussion

Wax gourd is a globally preferred vegetable due to its high nutritional value and health benefits. Among the different bioactive components, flavonoids, such as anthocyanins, flavonols, and flavan-3-ols, in the fruit have antiangiogenetic and anticancer properties (Jiang et al., 2015). Much effort has been made to elucidate the flavonoid biosynthetic pathway and identify the regulatory factors. A previous study showed that grape (*Vitis vinifera* L.) adapted to intense light by increasing the expression of flavonoid biosynthetic genes in the skin, leading to increased anthocyanin, proanthocyanidin, and flavonol content (Dobre et al., 2014). In litchi (*Litchi chinensis*), fruit bagging inhibited anthocyanin accumulation and biosynthetic genes encoding CHS, CHI, F3H, dihydroflavonol 4-reductase (DFR), anthocyanin synthase (ANS), and UFGT (Koyama et al., 2012). In this study, the flavonoid content was high in the EMS-induced mutant *hfc12*, indicating the existence of regulatory factors for flavonoid content in wax gourd. Metabolites, including flavonoid carbonosides, flavonols, isoflavones, flavanols, dihydroflavonol, and chalcones, were found to be differentially accumulated in the *hfc12*. Detailed analysis revealed that the DAFs and DEGs between the ‘BWT’ and *hfc12* were significantly enriched in the flavonoid pathway (**Figure 2**, **Figure 3**). These observations collectively suggested that various DAFs and DEGs have related roles, and transcription factors may potentially coordinate the synthesis of flavonoids in wax gourd.

Fine-mapping identified a typical two-component system (TCS) PRR transcription factor *BhiPRR6* on chromosome 12 of wax gourd, with a C-T nonsynonymous mutation in the positive chain CDS region (+), in the *hfc12* with higher levels of flavonoids. The candidate gene *BhiPRR6* was highly homologous to *APRR6* of *Arabidopsis*. The TCS signaling is an important mechanism regulating various pathways in prokaryotes and eukaryotes (Wei et al., 2011). This pathway usually consists of three signal elements: histidine kinases (HKs), response regulators (RRs), and histidine phosphate transfers (HPs) (Stock et al., 2000). The HK protein could be got phosphorylated, and this phosphate group gets continuously transferred to the conserved Asp residues in the RR receptor domain (Hwang et al., 2002). Phosphorylated RR protein directly or indirectly regulates the activity of downstream genes. The HP protein acts as a bridge in the phosphate transfer between HK and RR (Grefen and Harter, 2004). These response modifiers are divided into three subfamilies: type-A RRs with only receptor domains, type-B RRs with receptor domains fused to DNA binding domains, and PRR with atypical receptor domains (Huo et al., 2020). Type-B RRs bind to multiple cis-elements in the promoters of the target genes, such as mitogen-activated protein kinase (MAPK), or other regulatory genes, thereby participating in diverse regulatory responses (Wurgler-Murphy and Saito, 1997). The members of the PRR family have significant sequence similarity with the RRs in the putative receptor domain. They do not have a conserved D-D-K motif in the receptor domain and lack conserved Asp residues but can be used as the final product of two-component phosphorylation in plants. In this study, as **Figure 1** and **Figure S1B** shown, *BhiPRR6* was the highest expressed in fruit, and the flavonoid content of the *hfc12* was significantly higher than that of the WT, indicating the importance of *BhiPRR6* in regulating flavonoid biosynthesis in wax gourd.

Several factors, such as light, regulate flavonoid synthesis. Higher plants use sensory photoreceptors to accurately perceive light from UV-B to far-red wavelengths and chlorophyll and carotenoids of the photocomplex in photosynthesis (Casal, 2013). One of the most important is the superfamily of plant pigments, including photoreceptors (PHYA, PHYB, PHYC, PHYD, PHYE) that absorb red/far-red light and leuco pigments (CRY1, CRY2, CRY3) and photosensitive proteins (PHOT1, PHOTO2) (Wagner et al., 2005). In this study, the flavonoid content of the *hfc12* significantly increased, accompanied by a substantially lower chlorophyll a and chlorophyll b, indicating a close association between flavonoid synthesis and light pigments (**Figure 1**). Various transcription factors through differential expression regulate the biosynthesis of different flavonoids in response to specific light wavelengths (Zoratti et al., 2014). Moreover, in higher plants, the light pigments may be a His protein kinase (HKs). The light signals use the light-regulated phosphorous transfer mechanism, probably using a separate receptor protein RR as an intermediate (Huq et al., 2010). Therefore, HPs and RRs play essential roles in light signaling (Yeh et al., 1997). In *Arabidopsis*, *ARR4* transcription factor acts as a signaling module in the light signal transduction pathway (Haberer and Kieber, 2002). A study using GBS-based BSA-seq analysis in pepper identified a candidate gene *CaPRR2* related to plastid development and pigment biosynthesis, affecting the levels of chlorophyll a and chlorophyll b and controlling the color of fruit (Lee et al., 2020).

Furthermore, a typical MYB DNA-binding domain was identified in the candidate gene *BhiPRR6* sequence, and the mutation occurred in this region, indicating it has a function was similar with the MYB transcription factor. Studies have shown that MYB transcription factors coordinate and regulate flavonoid structural genes by activating or inhibiting their expression. MYB transcription factors related to flavonoid biosynthesis have been identified in various plants, with few responsive to light. In mango (*Mangifera indica* L.), *MiMYB1* at the transcript level regulates the light-dependent red coloration of fruit skin (Kanzaki et al., 2020). Moreover, the MYB transcription factor directly interacts with the MYB recognition element (MRE) in the promoter region of the structural genes (Yao et al., 2017). Studies have also reported that MRE is essential for light-induced expression of structural flavonoid genes, such as *CHS* (Takos et al., 2006). Typical MRE *cis*-elements were found in the promoter region of the candidate gene *BhiPRR6*. Other MYB typical *cis*-elements also were found in the promoter region of *BhiPRR6*, such as MRE, MYB, MYB-like sequence, MYC, MYB-binding site, and Myc. Subsequent structural analysis revealed the typical TCS and MYB-like functions of *BhiPRR6* in regulating the light-regulated pathway, leading to changes in flavonoid content of wax gourd.

Subsequently, a co-expression network was constructed using BhiPRR6 as hub gene to find why it can regulate the synthesis of flavonoids in wax gourd and leads to the change of peel color (**Figure 5**). Interestingly, many flavonoid-related structural genes were identified as the co-expressed genes, such as *Bhi4CL* and *BhiUFGT*; *4CL* forms the backbone unit for flavonoids, and *UFGT* acts in the last step of flavonoid biosynthesis for glycosylation modification (Jaakola, 2013). The co-expression plot also indicated the co-expression of *6LHCA6*, *MYB1R1*, and *WD40* with the hub gene *BhiPRR6*. Among them, MYB-bHLH-WD40 ternary complex (MBW complex) is a typical representative to positively regulate the biosynthesis of anthocyanins and flavonoids (Xu et al., 2021). The expression pattern of the co-expressed genes showed consistency with that of *BhiPRR6* and its associated target genes, indicating that they are similarly regulated in this *hfc12* (**Figure S2**). These observations in the *hfc12* suggest that *BhiPRR6* binds to the co-expressed genes through various *cis*-elements on the promoter. The MYB DNA-binding domain of *BhiPRR6* may regulate the conduction of light signals and the function of downstream genes and thereby inhibit flavonoid biosynthesis in wax gourd. In summary, it is collectively indicated that *BhiPRR6* and the co-expressed genes are highly expressed during the later stages of fruit development, inhibiting the transmission of light signals and leading to the reduction in flavonoids, which in turn leads to changes in peel color. This study provided new insights into the synthesis and regulation of flavonoids in wax gourd fruits and laid the foundation for breeding wax gourd varieties with higher levels of flavonoids.

## Data availability statement

The original contributions presented in the study are publicly available. This data can be found here: NCBI BioProject: PRJNA781301.

## Author contributions

LX, JW and FL: conceptualization, investigation, writing – review and editing, and preparation. JW: formal analysis, writing – original draft, and writing – original draft. BM, YC, LP, WX, and YL: investigation, formal analysis, and methodology. XS and CX: conceptualization, resources, supervision, funding acquisition, project administration, and writing – review and editing. All authors contributed to the article and approved the submitted version.

## Funding

This work was financed by National Natural Science Foundation of China (Grant No.32102398). The authors are grateful to Professor Xuexiao Zou from Hunan Agricultural University for providing the experimental environment and critical comments of this manuscript.

## Conflict of interest

The authors declare that the research was conducted in the absence of any commercial or financial relationships that could be construed as a potential conflict of interest.

## Publisher’s note

All claims expressed in this article are solely those of the authors and do not necessarily represent those of their affiliated organizations, or those of the publisher, the editors and the reviewers. Any product that may be evaluated in this article, or claim that may be made by its manufacturer, is not guaranteed or endorsed by the publisher.

## Supplementary material

Supplementary Figure 1
**(A)**. PCA plot of Metabonomic analysis. **(B)**. Transcript levels of *BhiPRR6* in flower, fruit, leaf, root, and stem of wax gourd. **(A)**. RNA-seq analysis. **(C)**. Soft threshold filter plot. The vertical axis on the left represented the square of the correlation coefficient between log(k) and log(p(k)) in the corresponding network. The higher the square of the correlation coefficient, the closer the network was to the distribution without network scale. The vertical axis of the right figure represented the mean value of all gene adjacency functions in the corresponding gene module. This study was used 7 as the soft threshold. **(D)**. Co-express network-level cluster analysis. The branches and different colors of the clustering tree are used to indicate different gene modules. **(E)**. GO enrichment analysis of co-expressed genes. Blue bars for cellular component, green bars for molecular function, and red bars for biological process. **(F)**. KEGG analysis of co-expressed genes.

Supplementary Figure 2 The expression heatmap of *BhiPRR6* (red color) and all highly co-expressed genes by RNA-seq analysis. All samples were collected at 40 DAP peels of wax gourd.

## References

[B1] Almeida J. D’Amico. E. Preuss A. F. Carbone. Vos C. Deiml B. Mourgues F.. (2007). Characterization of major enzymes and genes involved in flavonoid and proanthocyanidin biosynthesis during fruit development in strawberry (*Fragaria xananassa*). Arch. Biochem. Biophys. 465, 61–71. doi: 10.1016/j.abb.2007.04.040 17573033

[B2] Azuma A. Hiroshi Y. Yoshiko K. Shozo K. (2012). Flavonoid biosynthesis-related genes in grape skin are differentially regulated by temperature and light conditions. Planta 236, 1067–1080. doi: 10.1007/s00425-012-1650-x 22569920

[B3] Baranac J. M. Petranovi N. A. Dimitri-Markovi J. M. (1997). Spectrophotometric study of anthocyan copigmentation reactions. 2. Malvin nonglycosidized flavone quercetin. J. Agr. Food Chem. 45, 1698–1700. doi: 10.1021/jf9606114

[B4] Bolger A. M. Lohse M. Usadel B. (2014). Usadel, trimmomatic: a flexible trimmer for illumina sequence data. Bioinformatics 30, 2114–2120. doi: 10.1093/bioinformatics/btu170 24695404 PMC4103590

[B5] Brown J. Meg P. Lee A. M. C. (2017). McCue, FQC dashboard: integrates FastQC results into a web-based, interactive, and extensible FASTQ quality control tool. Bioinformatics 33, 3137–3139. doi: 10.1093/bioinformatics/btx373 28605449 PMC5870778

[B6] Casal J. J. (2013). Photoreceptor signaling networks in plant responses to shade. Ann. Rev. Plant Biol. 64, 403. doi: 10.1146/annurev-arplant-050312-120221 23373700

[B7] Chen C. Zhou G. Chen J. Liu X. Lu X. Chen H.. (2021). Integrated metabolome and transcriptome analysis unveils novel pathway involved in the formation of yellow peel in cucumber. Int. J. Mol. Sci. 22, 1494. doi: 10.3390/ijms22031494 33540857 PMC7867363

[B8] Dobre C. Toma E. Lucian I. Lavinia M. B. (2014). *Benincasa hispida* (wax gourd) technological characteristics and biological proprieties. Rom. Biotech. Lett. 19, 9504–9509.

[B9] Feder A. Burger J. Gao S. Lewinsohn E. Katzir N. Schaffer A. A.. (2015). A kelch domain-containing f-box coding gene negatively regulates flavonoid accumulation in muskmelon. Plant Physiol. 169, 1714–1726. doi: 10.1104/pp.15.01008 26358418 PMC4634078

[B10] Grefen C. Harter K. (2004). Plant two-component systems: Principles, functions, complexity and cross talk. Planta 219, 733–742. doi: 10.2307/23388587 15232695

[B11] Haberer G. Kieber J. J. (2002). New insights into a classic phytohormone. Plant Physiol. 128, 354–362. doi: 10.1104/pp.010773 11842139 PMC1540208

[B12] Hichri I. Barrieu F. Bogs J. Kappel C. Delrot S. Lauvergeat V. (2011). Recent advances in the transcriptional regulation of the flavonoid biosynthetic pathway. J. Exp. Bot. 62, 2465. doi: 10.1093/jxb/erq442 21278228

[B13] Hu D. G. Li Y. Y. Zhang Q. Y. Li M. Sun C. H. Yu J. Q.. (2017). The R2R3-MYB transcription factor *MdMYB73* is involved in malate accumulation and vacuolar acidification in apple. Plant J. 91, 443–454. doi: 10.1111/tpj.13579 28423209

[B14] Huo R. Liu Z. N. Yu X. L. Li Z. Y. (2020). The interaction network and signaling specificity of two-component system in *Arabidopsis* . Int. J. Mol. Sci. 21 (14), 4898. doi: 10.3390/ijms21144898 PMC7402358 32664520

[B15] Huq E. Al-Sady B. Quail P. H. (2010). Nuclear translocation of the photoreceptor phytochrome b is necessary for its biological function in seedling photomorphogenesis. Plant J. 35, 660–664. doi: 10.1046/j.1365-313X.2003.01836.x 12940958

[B16] Hwang I. Chen H. C. Sheen J. (2002). Two-component signal transduction pathways in *Arabidopsis* . Plant Physiol. 129, 500–515. doi: 10.1104/pp.005504 12068096 PMC161668

[B17] Imran M. Abdur R. Abu-Izneid T. (2019). Luteolin, a flavonoid, as an anticancer agent: A review. BioMed. Pharmacother. 112, 108612. doi: 10.1016/j.biopha.2019.108612 30798142

[B18] Jaakola L. (2013). New insights into the regulation of anthocyanin biosynthesis in fruits. Trends Plant Sci. 18 (9), 477–483. doi: 10.1016/j.tplants.2013.06.003 23870661

[B19] Jaakola L. Hohtola A. (2010). Effect of latitude on flavonoid biosynthesis in plants. Plant Cell Environ. 33, 1239–1247. doi: 10.1111/j.1365-3040.2010.02154.x 20374534

[B20] Jaakola L. Poole M. Jones M. O. (2019). A SQUAMOSA MADS box gene involved in the regulation of anthocyanin accumulation in bilberry fruits. Plant Physiol. 153, 1619–1629. doi: 10.1104/pp.110.158279 PMC2923880 20566708

[B21] Jiang B. Liu W. R. Xie D. S. Peng Q. W. He X. M. Lin Y. E.. (2015). High-density genetic map construction and gene mapping of pericarp color in wax gourd using specific-locus amplified fragment (SLAF) sequencing. BMC Genomics 16, 1035. doi: 10.1186/s12864-015-2220-y 26647294 PMC4673774

[B22] Kanzaki S. Ichihi A. Tanaka Y. Fujishige S. Shimizu K. (2020). The R2R3-MYB transcription factor *MiMYB1* regulates light dependent red coloration of 'Irwin' mango fruit skin. Scientia Hortic. 272, 109567. doi: 10.1016/j.scienta.2020.109567

[B23] Koyama K. Ikeda H. Poudel P. R. Goto-Yamamoto N. (2012). Light quality affects flavonoid biosynthesis in young berries of Cabernet sauvignon grape. Phytochemistry 78, 54–64. doi: 10.1016/j.phytochem.2012.02.026 22455871

[B24] Kurlovs A. H. Snoeck S. Kosterlitz O. Leeuwen T. Clark R. M. (2019). Trait mapping in diverse arthropods by bulked segregant analysis. Curr. Opin. Insect Sci. 36, 57–65. doi: 10.1016/j.cois.2019.08.004 31499416

[B25] Langfelder P. Horvath S. (2008). WGCNA: An r package for weighted correlation network analysis. BMC Bioinf. 9, 559. doi: 10.1186/1471-2105-9-559 PMC2631488 19114008

[B26] Lee S. B. Kim J. E. Kim H. T. Lee J. M. Kim B. S. Lee J. M. (2020). Genetic mapping of the c1 locus by GBS-based BSA-seq revealed pseudo-response regulator 2 as a candidate gene controlling pepper fruit color. Theor. Appl. Genet. 133, 1897–1910. doi: 10.1007/s00122-020-03565-5 32088729

[B27] Li Z. Peng R. Yao Q. (2021). *SlMYB14* promotes flavonoids accumulation and confers higher tolerance to 2,4,6-trichlorophenol in tomato. Plant Sci. 303, 110796. doi: 10.1016/j.plantsci.2020.110796 33487333

[B28] Li Y. Wen C. Weng Y. (2013). Fine mapping of the pleiotropic locus b for black spine and orange mature fruit color in cucumber identifies a 50 kb region containing a *R2R3-MYB* transcription factor. Theor. Appl. Gene1. 26 (8), 2187–2196. doi: 10.1007/s00122-013-2128-3 23689749

[B29] Love M. I. Huber W. Anders S. (2014). Moderated estimation of fold change and dispersion for RNA-seq data with DESeq2. Genome Biol. 15, 550. doi: 10.1186/s13059-014-0550-8 25516281 PMC4302049

[B30] Martens S. Preuss A. Matern U. (2010). Multifunctional flavonoid dioxygenases: flavonol and anthocyanin biosynthesis in *Arabidopsis thaliana* l. Phytochemistry 71, 1040–1049. doi: 10.1016/j.phytochem.2010.04.016 20457455

[B31] Pang Z. Q. Chong J. Li S. Z. Xia J. G. (2020). MetaboAnalystR 3.0: Toward an optimized workflow for global metabolomics. Metabolites 10 (5), 186. doi: 10.3390/metabo10050186 PMC7281575 32392884

[B32] Patro R. Duggal G. Love M. I. Irizarry R. A. Kingsford C. (2017). Salmon provides fast and bias-aware quantification of transcript expression. Nat. Methods 14, 417–419. doi: 10.1038/nmeth.4197 28263959 PMC5600148

[B33] Premathilake A. T. Ni J. B. Bai S. L. Tao R. Y. Ahmad M. Teng Y. W. (2020). R2R3-MYB transcription factor *PpMYB17* positively regulates flavonoid biosynthesis in pear fruit. Planta 252 (4), 59. doi: 10.1007/s00425-020-03473-4 32964301

[B34] Ramirez-Gonzalez R. H. Bonnal R. Caccamo M. Maclean D. (2012). Bio-samtools: Ruby bindings for SAMtools, a library for accessing BAM files containing high-throughput sequence alignments. Source Code Biol. Med. 7. doi: 10.1186/1751-0473-7-6 PMC3473260 22640879

[B35] Semagn K. Babu R. Hearne S. Olsen M. (2014). Single nucleotide polymorphism genotyping using kompetitive allele specific PCR (KASP): Overview of the technology and its application in crop improvement. Mol. Breed. 33, 1–14. doi: 10.1007/s11032-013-9917-x

[B36] Shi Q. Du J. Zhu D. Li X. Li X. (2020). Metabolomic and transcriptomic analyses of anthocyanin biosynthesis mechanisms in the color mutant *Ziziphus jujuba* cv. Tailihong. J. Agr. Food Chem. 68, 15186–15198. doi: 10.1021/acs.jafc.0c05334 33300333

[B37] Smoot M. E. Ono K. Ruscheinski J. Wang P. L. Ideker T. (2011). Cytoscape 2.8: new features for data integration and network visualization. Bioinformatics 27, 431–432. doi: 10.1093/bioinformatics/btq675 21149340 PMC3031041

[B38] Sreenivas K. M. Chaudhari K. Lele S. S. (2011). Ash gourd peel wax: Extraction, characterization, and application as an edible coat for fruits. Food Sci. Biotechnol. 20, 383–387. doi: 10.1007/s10068-011-0054-1

[B39] Stock A. M. Robinson V. L. Goudreau P. N. (2000). Two-component signal transduction. Annu. Rev. Biochem. 69, 183–215. doi: 10.1146/annurev.biochem.69.1.183 10966457

[B40] Sudheeran P. K. Sela N. Carmeli-Weissberg M. Ovadia R. Alkan N. (2021). Induced defense response in red mango fruit against colletotrichum gloeosporioides. Hortic. Res. 8, 17. doi: 10.1038/s41438-020-00452-4 33423039 PMC7797005

[B41] Takagi H. Abe A. Yoshida K. Kosugi S. Natsume S. Mitsuoka C. (2013). QTL-seq: Rapid mapping of quantitative trait loci in rice by whole genome resequencing of DNA from two bulked populations. Plant J. 74, 174–183. doi: 10.1111/tpj.12105 23289725

[B42] Takos A. M. Jaffé F. W. Jacob S. R. Bogs J. Robinson S. P. Walker A. R. (2006). Light-induced expression of a *MYB* gene regulates anthocyanin biosynthesis in red apples. Plant Physiol. 142, 1216–1232. doi: 10.1104/pp.106.088104 17012405 PMC1630764

[B43] Tomás-Barberán F. A. Gil M. I. Cremin P. Waterhouse A. L. Hess-Pierce B. Kader A. A. (2001). HPLC–DAD–ESIMS analysis of phenolic compounds in nectarines, peaches, and plums. J. Agr. Food Chem. 49, 4748–4760. doi: 10.1021/jf0104681 11600017

[B44] Voorrips R. E. (2002). MapChart: Software for the graphical presentation of linkage maps and QTLs. J. Hered. 93, 77–78. doi: 10.1093/jhered/93.1.77 12011185

[B45] Wagner J. R. Brunzelle J. S. Forest K. T. Vierstra R. D. (2005). A light-sensing knot revealed by the structure of the chromophore-binding domain of phytochrome. Nature 438, 325–331. doi: 10.1038/nature04118 16292304

[B46] Wei Y. Z. Hu F. C. Hu G. B. Li X. J. Huang X. M. Wang H. C.. (2011). Differential expression of anthocyanin biosynthetic genes in relation to anthocyanin accumulation in the pericarp of litchi chinensis sonn. PloS One 6, e19455. doi: 10.1371/journal.pone.0019455 21559331 PMC3084873

[B47] Winkel-Shirley B. (2002). Biosynthesis of flavonoids and effects of stress. Curr. Opin. Plant Bio 5, 218–223. doi: 10.1016/S1369-5266(02)00256-X 11960739

[B48] Wurgler-Murphy S. M. Saito H. (1997). Two-component signal transducers and MAPK cascades. Trends Biochem. Sci. 22, 172–176. doi: 10.1016/S0968-0004(97)01036-0 9175476

[B49] Xie D. Xu Y. Wang J. Liu W. Zhang Z. (2019). The wax gourd genomes offer insights into the genetic diversity and ancestral cucurbit karyotype. Nat. Commun. 10, 5158. doi: 10.1038/s41467-019-13185-3 31727887 PMC6856369

[B50] Xu P. Wu L. Cao M. Ma C. Xiao K. Li Y.. (2021). Identification of MBW complex components implicated in the biosynthesis of flavonoids in woodland strawberry. Front. Plant Sci. 12. doi: 10.3389/fpls.2021.774943 PMC8606683 34819941

[B51] Yan H. Pei X. Zhang H. Li X. Zhang X. Zhao M.. (2021). MYB-mediated regulation of anthocyanin biosynthesis. Int. J. Mol. Sci. 22, 3103. doi: 10.3390/ijms22063103 33803587 PMC8002911

[B52] Yao G. Ming M. Allan A. C. Chao G. Li L. Xiao W.. (2017). Map-based cloning of the pear gene *MYB114* identifies an interaction with other transcription factors to coordinately regulate fruit anthocyanin biosynthesis. Plant J. 92, 437–451. doi: 10.1111/tpj.13666 28845529

[B53] Yeh K. C. Wu S. H. Murphy J. T. Lagarias J. C. (1997). A cyanobacterial phytochrome two-component light sensory system. Science 277, 1505–1508. doi: 10.1126/science.277.5331.1505 9278513

[B54] Yuan F. Yang H. Xue Y. Kong D Ye R Li C. (2014). OSCA1 mediates osmotic-stress-evoked Ca2+ increases vital for osmosensing in arabidopsis. Nature 514 (7522), 367. doi: 10.1038/nature13593 25162526

[B55] Yu G. Wang L. G. Han Y. He Q. Y. (2012). ClusterProfiler: an r package for comparing biological themes among gene clusters. Omics 16, 284–287. doi: 10.1089/omi.2011.0118 22455463 PMC3339379

[B56] Zoratti L. Karppinen K. Escobar A. L. Häggman H. Jaakola L. (2014). Light-controlled flavonoid biosynthesis in fruits. Front. Plant Sci. 5. doi: 10.3389/fpls.2014.00534 PMC4191440 25346743

