# Supplementary material for: Integrated analysis of multi-omics and fine-mapping reveals a candidate gene regulating pericarp color and flavonoids accumulation in wax gourd (Benincasa hispida)
Source: Front Plant Sci. 2022 Sep 26;13:1019787. doi: 10.3389/fpls.2022.1019787 (PMC9549291; doi:10.3389/fpls.2022.1019787)
Supplement: Supplementary file 1 [file Table_1.docx]

Table S1. Quality assessment of RNA-seq data

| **Sample** | **RAW_READS** | **RAW_BASES** | **READ_LENGTH** | **RawQ20** | **RawQ30** |
| --- | --- | --- | --- | --- | --- |
| GM-1 | 25298148 | 7,589,444,400 | 150;150 | 97.79;96.15 | 94.01;91.16 |
| GM-2 | 20272890 | 6,081,867,000 | 150;150 | 97.64;95.93 | 93.75;90.87 |
| GM-3 | 20554068 | 6,166,220,400 | 150;150 | 97.89;96.80 | 94.31;92.33 |
| GW-1 | 21964727 | 6,589,418,100 | 150;150 | 97.62;95.94 | 93.51;90.63 |
| GW-2 | 20726819 | 6,218,045,700 | 150;150 | 97.98;96.76 | 94.44;92.38 |
| GW-3 | 22985721 | 6,895,716,300 | 150;150 | 97.97;96.67 | 94.48;92.17 |
