# Supplementary material for: Integrated analysis of multi-omics and fine-mapping reveals a candidate gene regulating pericarp color and flavonoids accumulation in wax gourd (Benincasa hispida)
Source: Front Plant Sci. 2022 Sep 26;13:1019787. doi: 10.3389/fpls.2022.1019787 (PMC9549291; doi:10.3389/fpls.2022.1019787)
Supplement: Supplementary file 3 [file Table_3.docx]

Table S3 Gene mapping via BSA-seq

| Sequencing data ID | Number of Raw reads | Number of Raw reads | Read length | GC% | Bases Q30% | Genome_len | Base_number_in_Depth | Coverage | Average_Depth |
| --- | --- | --- | --- | --- | --- | --- | --- | --- | --- |
| ‘BWT’(WT) | 76,087,882 | 11,413,182,300 | 150 | 35.68 | 94.36 | 912,951,626 | 872,774,967 | 0.9560 | 10.47 |
| *hfc12* | 61,626,036 | 9,643,905,400 | 150 | 36.18 | 91.36 | 912,951,626 | 875,818,057 | 0.9593 | 18.92 |
| Dominant mixed pool | 173,094,036 | 25,964,105,400 | 150 | 37.45 | 91.18 | 912,951,626 | 873,454,487 | 0.9567 | 10.05 |
| Recessive mixed pool | 135,134,612 | 20,270,191,800 | 150 | 36.27 | 90.99 | 912,951,626 | 875,754,265 | 0.9593 | 16.82 |

Note: 1. Sequencing data ID: Samples name; 2. Number of Raw reads: the number of reads of raw sequencing data; 3. Number of Raw Base: The output of raw sequencing data, the number of reads is multiplied by the length of the sequencing sequence, with bp as the unit; 4. Genome_len: reference genome ‘*B227*’ reads length. 5. GC_Rate (%): the content of bases G and C; 6. Q30_Rate (%): the percentage of bases with a Phred value > 30 to the total bases; 7. Base_number_in_Depth: Total number of reads used for mapping; 8. Coverage: Coverage genome rate, the percentage of the number of reads compared to the reference genome to the total number of reads used for mapping; 9. Average depth: The average sequencing depth, the amounts of clean bases data divided by the size of the reference genome.
