# Supplementary material for: Integrated analysis of multi-omics and fine-mapping reveals a candidate gene regulating pericarp color and flavonoids accumulation in wax gourd (Benincasa hispida)
Source: Front Plant Sci. 2022 Sep 26;13:1019787. doi: 10.3389/fpls.2022.1019787 (PMC9549291; doi:10.3389/fpls.2022.1019787)
Supplement: Supplementary file 4 [file Table_4.docx]

Table S4 Primer sequences

| Gene | Left primer (5’-3’) | Right primer (5’-3’) | Center Primer (SNP 5’-3’) |
| --- | --- | --- | --- |
| *BhiPRR6* | ATGTCCCCTGTGAAGGTTTTGCTC | ACAAAACAACAGTGTCCGCAAT | - |
| *BhiPRR6_qRT-PCR* | TTCAGCATGTCGGCTCAAAC | TCCTCCTATGGGAGCAGACT | - |
| *Bhi05G000810_qRT-PCR* | GGCTCTGATGGAGGTAGGTC | ACCTCCATGACCAGAACCAG | - |
| *Bhi06G000135_qRT-PCR* | GATTCGAACCCAGAGGTTGC | TCCAATTTCACCGTCAACCG | - |
| *Bhi10G001402_qRT-PCR* | TCTTCCATGCACACCAGTCT | TGAAGAACGCAGAAGCAGTG | - |
| *Bhi09G001849_qRT-PCR* | AGGATCGGGTAACAGCTACG | AGTCTAGAAGCCGCCATGTT | - |
| *Bhi03G000738_qRT-PCR* | GATCTCTCAGAGCGTGGTGA | AACGTGCAGCTTCCAATGTT | - |
| *Bhi05G000624_qRT-PCR* | GCATGCCACAATGGACAGAT | TCCCATCTTCACCCACCTTC | - |
| *Bhi11G001762_qRT-PCR* | GGTGAGTGAAGTGGGTCAGA | GTCCTGATCTTGGGTCACCA | - |
| *Bhi09G002071_qRT-PCR* | GTGTCCGCAAGAAGAGGTTC | AGAACGGCCAGCAAATCATC | - |
| *Bhi11G000031_qRT-PCR* | TCAAAGCGGGTCGAACAATC | CCTCAGTCCATGGAACACCT | - |
| *Bhi05G000029_qRT-PCR* | TTGCAAAGCTTCCACCGAAA | GTGGTTTGATCCGTCCACTG | - |
| *Actin_qRT-PCR* | ATGTTCACAACCACTGCCGA | GTCGAGCGCAACATAAGCAA | - |
| SNP12G24166107 | GAAGGTGACCAAGTTCATGCTAGTAGGAGTAGTGCTTAGCTCATAAA | GAAGGTCGGAGTCAACGGATTAGTAGGAGTAGTGCTTAGCTCATAAC | TGATTCGAGGTCAAACTTGAGACT |
| SNP12G25509139 | GAAGGTGACCAAGTTCATGCTCGATATTTAAGCTTCGAAAGGGAATTAG | GAAGGTCGGAGTCAACGGATTCGATATTTAAGCTTCGAAAGGGAATTAA | TTTTTAATCACAGGGAGCTGAAGC |
| SNP12G25877875 | GAAGGTGACCAAGTTCATGCTGCCTACGTTTGAAAATCTGCTTGC | GAAGGTCGGAGTCAACGGATTGCCTACGTTTGAAAATCTGCTTGT | TAACAGAAGGGAGGGATCCAAATC |
| SNP12G25961505 | GAAGGTGACCAAGTTCATGCTTTCATGCTATGAAAACTCTTTCCG | GAAGGTCGGAGTCAACGGATTTTCATGCTATGAAAACTCTTTCCA | ATTCAAACACATGTCCAGTCCATT |
| SNP12G26007829 | GAAGGTGACCAAGTTCATGCTAAGAACTCTCATTTGTGTTATGGAC | GAAGGTCGGAGTCAACGGATTAAGAACTCTCATTTGTGTTATGGAT | AGAGCGCATTTCAGCTTATGTTTT |
| SNP12G27913976 | GAAGGTGACCAAGTTCATGCTCCCGCCTATTCAGGGCTG | GAAGGTCGGAGTCAACGGATTCCCGCCTATTCAGGGCTA | CTGGTTCCCACTAGAGATGTCAAA |
| SNP12G28185295 | GAAGGTGACCAAGTTCATGCTTTGAAACCATCCAATACACAAAAATC | GAAGGTCGGAGTCAACGGATTTTGAAACCATCCAATACACAAAAATT | CGCTACAAAGAATTCCAAACCCTT |
| SNP12G30577665 | GAAGGTGACCAAGTTCATGCTAAGTTCTCTCACTTAATCATAACTCAC | GAAGGTCGGAGTCAACGGATTAAGTTCTCTCACTTAATCATAACTCAT | GGAAGGCTAAAGGAACATACAAGC |
| SNP12G35908349 | GAAGGTGACCAAGTTCATGCTCGTACTTGAGAAAGTAGTCTAAAGACC | GAAGGTCGGAGTCAACGGATTCGTACTTGAGAAAGTAGTCTAAAGACT | GCATGCGATGTGTAAATAGCAGAT |
