# Supplementary figures and images for: Integrated analysis of multi-omics and fine-mapping reveals a candidate gene regulating pericarp color and flavonoids accumulation in wax gourd (Benincasa hispida)
Source: Front Plant Sci. 2022 Sep 26;13:1019787. doi: 10.3389/fpls.2022.1019787 (PMC9549291; doi:10.3389/fpls.2022.1019787)

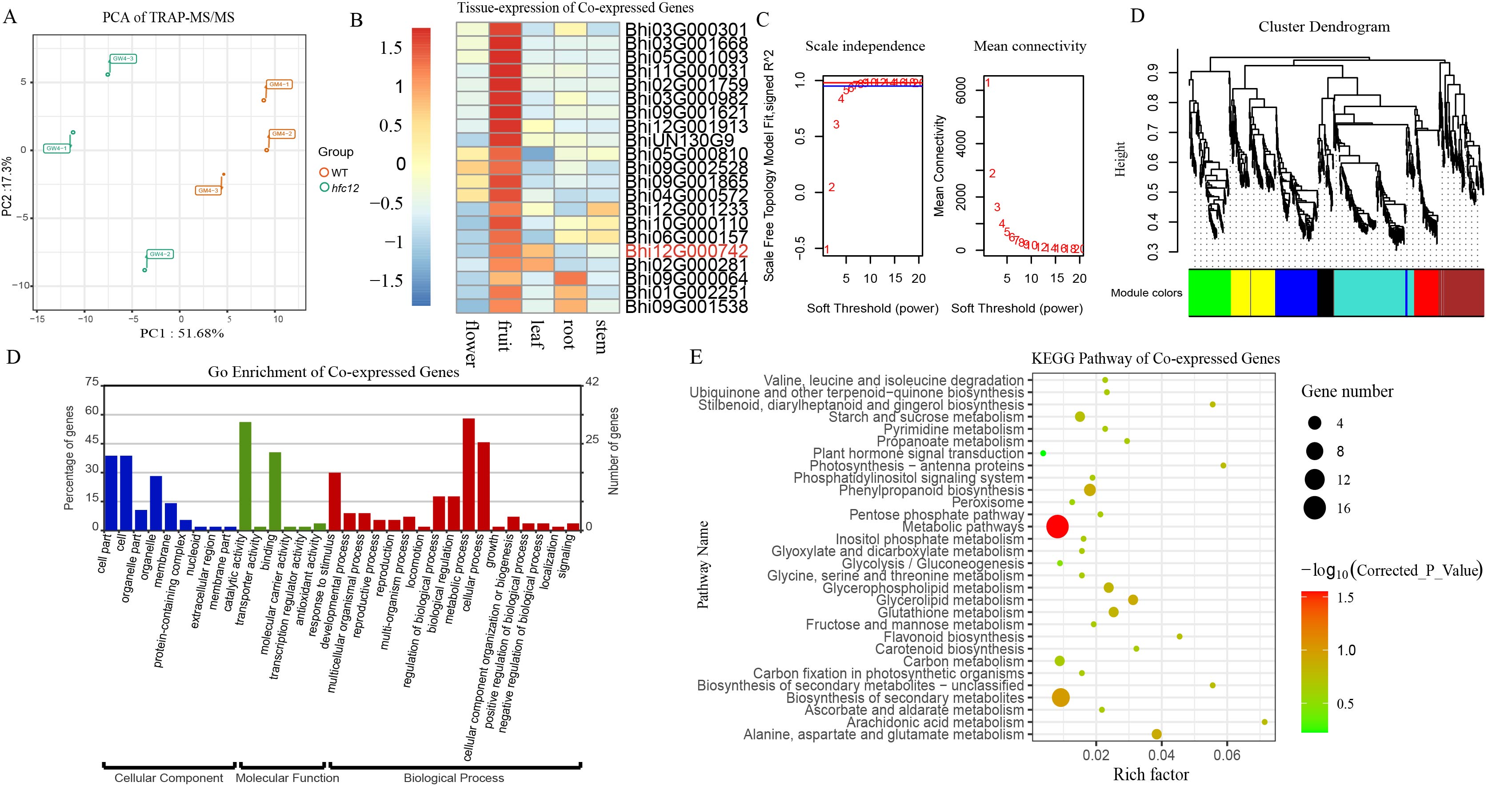

Supplement: Supplementary Figure 1 — (A). PCA plot of Metabonomic analysis. (B). Transcript levels of BhiPRR6 in flower, fruit, leaf, root, and stem of wax gourd. (A). RNA-seq analysis. (C). Soft threshold filter plot. The vertical axis on the left represented the square of the correlation coefficient between log(k) and log(p(k)) in the corresponding network. The higher the square of the correlation coefficient, the closer the network was to the distribution without network scale. The vertical axis of the right figure represented the mean value of all gene adjacency functions in the corresponding gene module. This study was used 7 as the soft threshold. (D). Co-express network-level cluster analysis. The branches and different colors of the clustering tree are used to indicate different gene modules. (E). GO enrichment analysis of co-expressed genes. Blue bars for cellular component, green bars for molecular function, and red bars for biological process. (F). KEGG analysis of co-expressed genes. [file Image_1.jpeg]

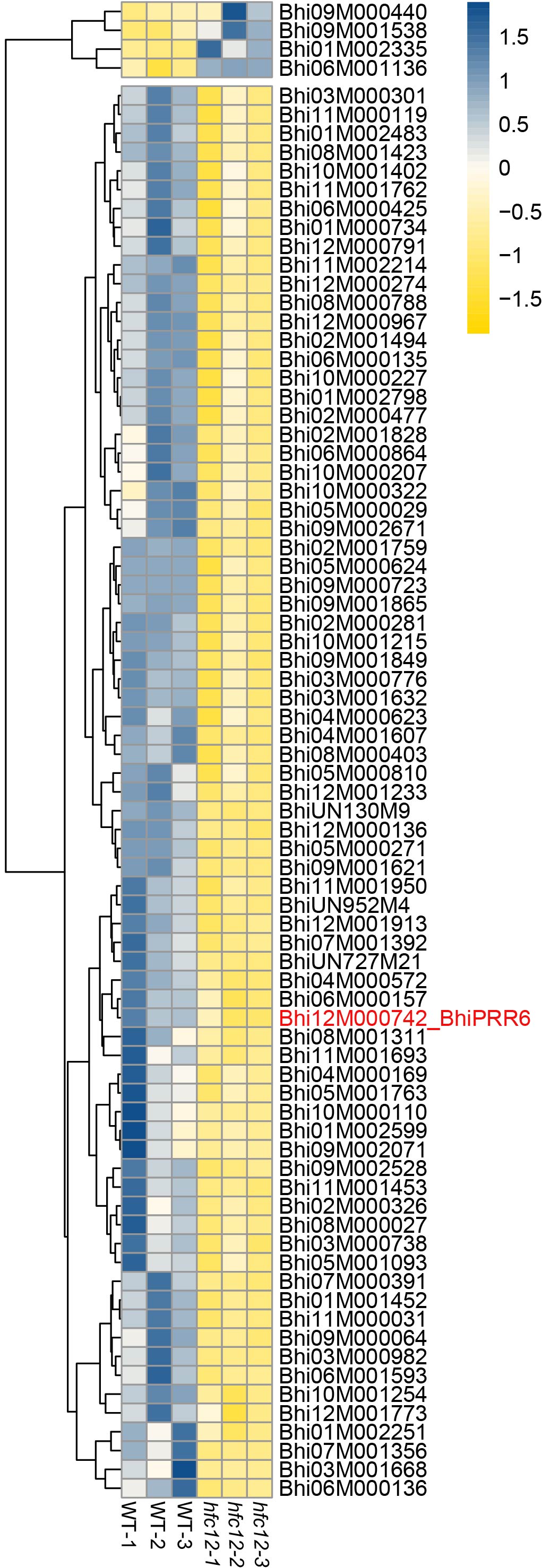

Supplement: Supplementary Figure 2 — The expression heatmap of BhiPRR6 (red color) and all highly co-expressed genes by RNA-seq analysis. All samples were collected at 40 DAP peels of wax gourd. [file Image_2.jpeg]
